# Diaqua­bis­(1,10-phenanthroline-κ^2^ *N*,*N*′)cadmium sulfate hexa­hydrate

**DOI:** 10.1107/S1600536811043194

**Published:** 2011-10-29

**Authors:** Kai-Long Zhong

**Affiliations:** a Department of Applied Chemistry, Nanjing College of Chemical Technology, Nanjing 210048, People’s Republic of China

## Abstract

The title compound, [Cd(C_12_H_8_N_2_)_2_(H_2_O)_2_]SO_4_·6H_2_O, was obtained unexpectedly during an attempt to synthesize a cadmium complex with bidentate bridging sulfate ligands *via* hydro­thermal synthesis. The Cd^II^ metal ion is six-coordinated by two chelating 1,10-phenanthroline ligands and two water mol­ecules, resulting in a distorted octa­hedral geometry for the metal ion. The two chelating N_2_C_2_ groups are almost perpendicular to each other [dihedral angle = 86.75 (2)°]. In the crystal, the [Cd(C_12_H_8_N_2_)_2_(H_2_O)_2_]^2+^ complex cations join with the sulfate anions through two O_water_—H⋯O_sulfate_ hydrogen bonds. These ion pairs are further inter­linked into a two-dimensional supermolecular structure *via* additional O—H⋯O hydrogen bonds.

## Related literature

For background to phenanthroline complexes, see: Zhong *et al.* (2006 ▶, 2009 ▶); Zhu *et al.* (2006 ▶); Ni *et al.* (2010 ▶); Zhong (2010 ▶); Cui *et al.* (2010 ▶). For related structures of six-coordinate cadmium complexes and background references, see: Yang *et al.* (2003 ▶); Lu *et al.* (2006 ▶); Zhong & Cui (2010 ▶). For standard bond lengths, see: Allen *et al.* (1987 ▶).
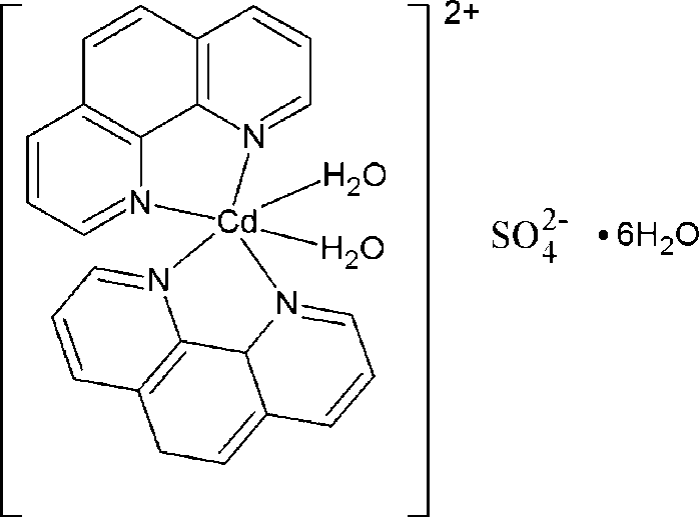


## Experimental

### 

#### Crystal data

[Cd(C_12_H_8_N_2_)_2_(H_2_O)_2_]SO_4_·6H_2_O
*M* *_r_* = 713.02
Triclinic, 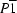

*a* = 10.344 (2) Å
*b* = 12.086 (2) Å
*c* = 13.331 (3) Å
α = 71.54 (3)°
β = 88.37 (3)°
γ = 69.37 (3)°
*V* = 1473.0 (7) Å^3^
*Z* = 2
Mo *K*α radiation
μ = 0.88 mm^−1^
*T* = 223 K
0.30 × 0.25 × 0.12 mm

#### Data collection

Rigaku Mercury CCD diffractometer
Absorption correction: multi-scan (*REQAB*: Jacobson, 1998 ▶) *T*_min_ = 0.741, *T*_max_ = 1.000
14255 measured reflections
6631 independent reflections
5729 reflections with *I* > 2σ(*I*)
*R*_int_ = 0.038

#### Refinement

*R*[*F* ^2^ > 2σ(*F* ^2^)] = 0.040
*wR*(*F* ^2^) = 0.082
*S* = 1.04
6631 reflections
427 parameters
63 restraints
H atoms treated by a mixture of independent and constrained refinement
Δρ_max_ = 0.56 e Å^−3^
Δρ_min_ = −0.54 e Å^−3^

### 

Data collection: *CrystalClear* (Rigaku, 2007 ▶); cell refinement: *CrystalClear*; data reduction: *CrystalClear*; program(s) used to solve structure: *SHELXS97* (Sheldrick, 2008 ▶); program(s) used to refine structure: *SHELXL97* (Sheldrick, 2008 ▶); molecular graphics: *XP* in *SHELXTL* (Sheldrick, 2008 ▶); software used to prepare material for publication: *SHELXTL*.

## Supplementary Material

Crystal structure: contains datablock(s) global, I. DOI: 10.1107/S1600536811043194/bq2310sup1.cif

Structure factors: contains datablock(s) I. DOI: 10.1107/S1600536811043194/bq2310Isup2.hkl

Additional supplementary materials:  crystallographic information; 3D view; checkCIF report

## Figures and Tables

**Table:** 

Cd1—O2*W*	2.252 (2)
Cd1—O1*W*	2.286 (2)
Cd1—N4	2.327 (2)
Cd1—N1	2.342 (2)
Cd1—N3	2.350 (2)
Cd1—N2	2.377 (2)

**Table:** 

O2*W*—Cd1—O1*W*	82.11 (8)
N4—Cd1—N3	71.63 (8)
N1—Cd1—N2	71.32 (8)

**Table 2:** Hydrogen-bond geometry (Å, °)

*D*—H⋯*A*	*D*—H	H⋯*A*	*D*⋯*A*	*D*—H⋯*A*
O1*W*—H1*WA*⋯O1	0.84 (2)	1.84 (2)	2.657 (3)	163 (3)
O2*W*—H2*WB*⋯O3	0.81 (3)	1.92 (3)	2.718 (3)	169 (4)
O1*W*—H1*WB*⋯O6*W*	0.85 (2)	1.91 (2)	2.739 (3)	163 (3)
O5*W*—H5*WA*⋯O2	0.77 (3)	2.08 (3)	2.816 (3)	161 (5)
O8*W*—H8*WA*⋯O1	0.85 (3)	1.88 (3)	2.729 (4)	175 (5)
O4*W*—H4*WA*⋯O2	0.87 (2)	1.99 (3)	2.805 (3)	157 (4)
O6*W*—H6*WB*⋯O5*W*	0.84 (2)	2.04 (3)	2.830 (4)	158 (4)
O7*W*—H7*WA*⋯O4	0.83 (3)	1.96 (3)	2.791 (4)	172 (6)
O7*W*—H7*WB*⋯O8*W*	0.82 (3)	2.16 (4)	2.901 (5)	151 (6)
O2*W*—H2*WA*⋯O3^i^	0.83 (4)	1.86 (4)	2.671 (3)	164 (3)
O3*W*—H3*WB*⋯O8*W*^ii^	0.77 (3)	2.29 (5)	2.907 (5)	137 (6)
O5*W*—H5*WB*⋯O4*W*^iii^	0.77 (3)	2.07 (3)	2.829 (4)	174 (5)
O6*W*—H6*WA*⋯O3*W*^iv^	0.78 (3)	2.03 (3)	2.773 (5)	160 (4)
O4*W*—H4*WB*⋯O7*W*^v^	0.79 (3)	2.03 (3)	2.806 (4)	168 (5)
O8*W*—H8*WB*⋯O3*W*^iv^	0.83 (3)	2.09 (3)	2.851 (5)	154 (5)

Symmetry codes: (i) 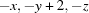; (ii) 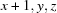; (iii) 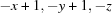; (iv) 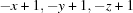; (v) 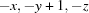.

**Fig. 1.**
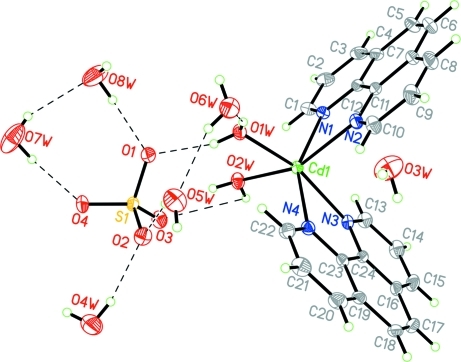
The asymmetric unit of (I), showing the atom-numbering scheme and with displacement ellipsoids drawn at the 30% probability level. The dashed lines represent O—H···O interactions.

**Fig. 2.**
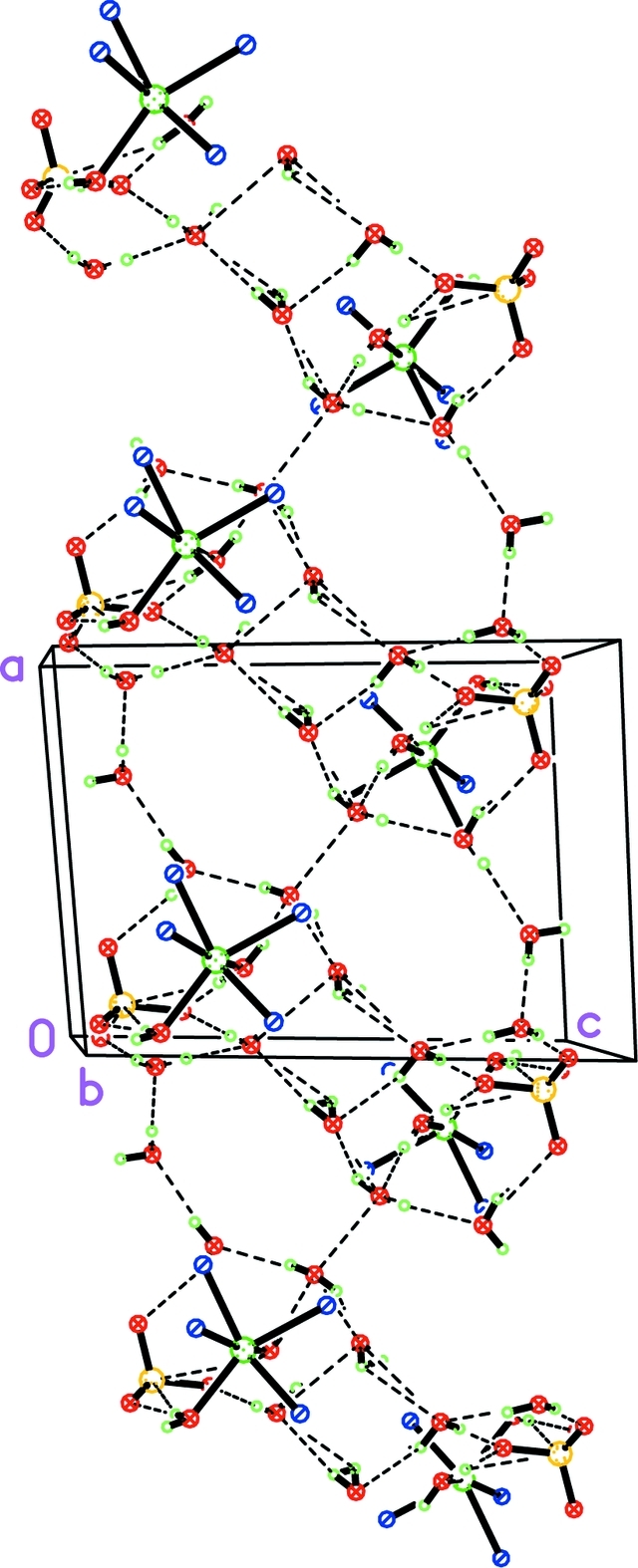
The packing viewed down the b axis. Dashed lines indicate hydrogen bonds. All H atoms and C atoms of phen have been omitted for clarity.

## supplementary crystallographic information

### Comment

Recently we have synthesized and reported many four-membered ring structural characteristics metal-Phen (1,10-phenanthroline) complexes with bidentate-chelating sulfate auxiliary ligand *via* a hydrothermal (solvothermal) reaction, such as cobalt complexes (Zhong *et al.*, 2006; Zhong, 2010), nickel complexes (Zhong *et al.*, 2009; Ni *et al.*, 2010), zinc complex (Cui *et al.*, 2010), and manganese complex (Zhu *et al.*, 2006). The title compound [Cd(C_12_H_8_N_2_)_2_(H_2_O)_2_]SO_4_.6H_2_O, (I) was obtained during an attempt to synthesize a four-membered ring structural characteristics Cd-complex with bidentate-chelating sulfate ligand by the similar route. Here we report the crystal structure of (I).

In the cation of (I), all bond lengths and angles are normal (Allen *et al.*, 1987). The Cd^2+^ metal ion has a distorted octahedral coordination environment composed of four N atoms from two chelating Phen ligands and two O atoms from two water molecules. The dihedral angle between the two chelating N_2_C_2_ groups is 86.75 (2) Å. The Cd—O bond distances [2.256 - 2.288 Å], the Cd—N bond distances [2.327 (2) - 2.377 (2) Å] and the N—Cd—N bite angles [71.32 (8)–71.63 (8)°] (Table 1.) are in good accord with those observed in many six-coordinate Cd-phen complexes [Cd(phen)_2_(H_2_O)_2_](C_4_H_2_O_4_).4H_2_O (Yang *et al.*, 2003), [CdSO_4_(C_12_H_8_N_2_)_2_].C_2_H_6_O_2_ (Lu *et al.*, 2006) and [CdSO_4_(C_12_H_8_N_2_)_2_].C_3_H_8_O_2_ (Zhong & Cui, 2010). The [Cd(C_12_H_8_N_2_)_2_(H_2_O)_2_]^2+^ complex cations and uncoordinated sulfate anion are connected by intermolecular O—H···O hydrogen bonds with the coordinated water molecules as donors (Fig. 1. and Table 2.). These units are further held together by typical O—H···O hydrogen bonding with uncoordinated water forming a two-dimensional hydrogen bond network (Fig. 2. and Table 2.).

### Experimental

0.2 mmol phen, 0.1 mmol 3CdSO_4_.8H_2_O and 2.0 ml water were mixed and placed in a thick Pyrex tube, which was sealed and heated to 413 K for 72 h, whereupon colorless block-shaped crystals of (I) were obtained.

### Refinement

The H atoms of phen were positioned geometrically and allowed to ride on their parent atoms, with C—H = 0.93 Å and *U*_iso_(H) = 1.2*U*_eq_(C). The H atoms of waters were located in difference map and then allowed to ride on their parent atoms, with O—H = 0.77 (3) - 0.87 (2)Å and *U*_iso_(H) =  1.5*U*_eq_(O).

### Crystal data

**Table:**

[Cd(C_12_H_8_N_2_)_2_(H_2_O)_2_]SO_4_·6H_2_O	*Z* = 2
*M**_r_* = 713.02	*F*(000) = 728
Triclinic, *P*1	*D*_x_ = 1.608 Mg m^−^^3^
Hall symbol: -p 1	Mo *K*α radiation, λ = 0.71073 Å
*a* = 10.344 (2) Å	Cell parameters from 7054 reflections
*b* = 12.086 (2) Å	θ = 3.0–27.5°
*c* = 13.331 (3) Å	µ = 0.88 mm^−^^1^
α = 71.54 (3)°	*T* = 223 K
β = 88.37 (3)°	Block, colorless
γ = 69.37 (3)°	0.30 × 0.25 × 0.12 mm
*V* = 1473.0 (7) Å^3^	

### Data collection

**Table:**

Rigaku Mercury CCD diffractometer	6631 independent reflections
Radiation source: fine-focus sealed tube	5729 reflections with *I* > 2σ(*I*)
Graphite Monochromator	*R*_int_ = 0.038
Detector resolution: 28.5714 pixels mm^-1^	θ_max_ = 27.5°, θ_min_ = 3.0°
ω scans	*h* = −11→13
Absorption correction: multi-scan (REQAB: Jacobson, 1998)	*k* = −12→15
*T*_min_ = 0.741, *T*_max_ = 1.000	*l* = −17→17
14255 measured reflections	

### Refinement

**Table:**

Refinement on *F*^2^	Primary atom site location: structure-invariant direct methods
Least-squares matrix: full	Secondary atom site location: difference Fourier map
*R*[*F*^2^ > 2σ(*F*^2^)] = 0.040	Hydrogen site location: inferred from neighbouring sites
*wR*(*F*^2^) = 0.082	H atoms treated by a mixture of independent and constrained refinement
*S* = 1.04	*w* = 1/[σ^2^(*F*_o_^2^) + (0.0339*P*)^2^] where *P* = (*F*_o_^2^ + 2*F*_c_^2^)/3
6631 reflections	(Δ/σ)_max_ = 0.002
427 parameters	Δρ_max_ = 0.56 e Å^−^^3^
63 restraints	Δρ_min_ = −0.54 e Å^−^^3^

### Special details

**Table:**

Geometry. All e.s.d.'s (except the e.s.d. in the dihedral angle between two l.s. planes) are estimated using the full covariance matrix. The cell e.s.d.'s are taken into account individually in the estimation of e.s.d.'s in distances, angles and torsion angles; correlations between e.s.d.'s in cell parameters are only used when they are defined by crystal symmetry. An approximate (isotropic) treatment of cell e.s.d.'s is used for estimating e.s.d.'s involving l.s. planes.
Refinement. Refinement of *F*^2^ against ALL reflections. The weighted *R*-factor *wR* and goodness of fit *S* are based on *F*^2^, conventional *R*-factors *R* are based on *F*, with *F* set to zero for negative *F*^2^. The threshold expression of *F*^2^ > σ(*F*^2^) is used only for calculating *R*-factors(gt) *etc*. and is not relevant to the choice of reflections for refinement. *R*-factors based on *F*^2^ are statistically about twice as large as those based on *F*, and *R*- factors based on ALL data will be even larger.

### Fractional atomic coordinates and isotropic or equivalent isotropic displacement parameters (Å^2^)

**Table:**

	*x*	*y*	*z*	*U*_iso_*/*U*_eq_	
Cd1	0.23865 (2)	0.943079 (18)	0.256252 (15)	0.02386 (8)	
N1	0.0916 (2)	1.0674 (2)	0.34727 (17)	0.0264 (5)	
N2	0.3565 (2)	0.9003 (2)	0.42255 (17)	0.0284 (5)	
N3	0.3139 (2)	1.1059 (2)	0.16124 (17)	0.0257 (5)	
N4	0.4515 (2)	0.8559 (2)	0.19554 (17)	0.0261 (5)	
C1	−0.0357 (3)	1.1479 (3)	0.3114 (2)	0.0340 (7)	
H1A	−0.0729	1.1533	0.2466	0.041*	
C2	−0.1168 (3)	1.2248 (3)	0.3657 (2)	0.0396 (8)	
H2A	−0.2062	1.2797	0.3378	0.047*	
C3	−0.0628 (3)	1.2183 (3)	0.4607 (3)	0.0410 (8)	
H3A	−0.1147	1.2700	0.4976	0.049*	
C4	0.0713 (3)	1.1334 (3)	0.5022 (2)	0.0346 (7)	
C5	0.1330 (4)	1.1184 (3)	0.6030 (2)	0.0461 (9)	
H5A	0.0839	1.1679	0.6426	0.055*	
C6	0.2605 (4)	1.0339 (4)	0.6410 (3)	0.0476 (9)	
H6A	0.2973	1.0251	0.7071	0.057*	
C7	0.3410 (3)	0.9572 (3)	0.5826 (2)	0.0353 (7)	
C8	0.4746 (4)	0.8676 (3)	0.6194 (2)	0.0450 (9)	
H8A	0.5143	0.8551	0.6858	0.054*	
C9	0.5462 (3)	0.7992 (3)	0.5591 (2)	0.0425 (8)	
H9A	0.6354	0.7409	0.5827	0.051*	
C10	0.4836 (3)	0.8180 (3)	0.4607 (2)	0.0357 (7)	
H10A	0.5331	0.7705	0.4198	0.043*	
C11	0.2847 (3)	0.9702 (3)	0.4822 (2)	0.0277 (6)	
C12	0.1462 (3)	1.0590 (3)	0.4422 (2)	0.0268 (6)	
C13	0.2470 (3)	1.2273 (3)	0.1431 (2)	0.0327 (7)	
H13A	0.1570	1.2536	0.1626	0.039*	
C14	0.3050 (4)	1.3174 (3)	0.0961 (2)	0.0408 (8)	
H14A	0.2545	1.4017	0.0846	0.049*	
C15	0.4364 (3)	1.2801 (3)	0.0676 (2)	0.0388 (8)	
H15A	0.4767	1.3390	0.0367	0.047*	
C16	0.5120 (3)	1.1517 (3)	0.0849 (2)	0.0300 (7)	
C17	0.6509 (3)	1.1059 (3)	0.0573 (2)	0.0377 (8)	
H17A	0.6948	1.1620	0.0261	0.045*	
C18	0.7190 (3)	0.9824 (3)	0.0760 (2)	0.0364 (8)	
H18A	0.8093	0.9547	0.0575	0.044*	
C19	0.6555 (3)	0.8938 (3)	0.1235 (2)	0.0286 (6)	
C20	0.7228 (3)	0.7645 (3)	0.1448 (2)	0.0392 (8)	
H20A	0.8137	0.7329	0.1286	0.047*	
C21	0.6545 (3)	0.6858 (3)	0.1891 (3)	0.0412 (8)	
H21A	0.6986	0.6001	0.2036	0.049*	
C22	0.5191 (3)	0.7339 (3)	0.2126 (2)	0.0331 (7)	
H22A	0.4732	0.6789	0.2416	0.040*	
C23	0.5182 (3)	0.9356 (2)	0.1510 (2)	0.0235 (6)	
C24	0.4456 (3)	1.0676 (3)	0.1316 (2)	0.0234 (6)	
O2W	0.0576 (2)	0.9861 (2)	0.14208 (17)	0.0336 (5)	
H2WA	0.019 (4)	1.053 (3)	0.094 (3)	0.050*	
H2WB	0.062 (4)	0.932 (3)	0.118 (3)	0.050*	
O1W	0.2142 (2)	0.75290 (19)	0.31802 (16)	0.0311 (5)	
H1WA	0.178 (3)	0.737 (3)	0.271 (2)	0.047*	
H1WB	0.274 (3)	0.687 (3)	0.361 (2)	0.047*	
O1	0.0996 (2)	0.6642 (2)	0.20075 (15)	0.0408 (5)	
O2	0.2611 (2)	0.63092 (19)	0.06966 (17)	0.0378 (5)	
O3	0.0709 (2)	0.82585 (18)	0.03451 (16)	0.0366 (5)	
O4	0.0277 (2)	0.6410 (2)	0.04110 (17)	0.0399 (5)	
O6W	0.3939 (3)	0.5180 (2)	0.43016 (19)	0.0463 (6)	
H6WB	0.417 (4)	0.482 (4)	0.385 (3)	0.069*	
H6WA	0.343 (4)	0.494 (4)	0.468 (3)	0.069*	
O5W	0.4613 (3)	0.4635 (3)	0.2396 (2)	0.0555 (7)	
H5WA	0.395 (3)	0.508 (4)	0.204 (3)	0.083*	
H5WB	0.523 (4)	0.448 (4)	0.207 (3)	0.083*	
O4W	0.2984 (3)	0.6079 (3)	−0.1330 (2)	0.0674 (8)	
H4WA	0.312 (5)	0.606 (4)	−0.069 (2)	0.101*	
H4WB	0.238 (4)	0.583 (5)	−0.135 (3)	0.101*	
O3W	0.8044 (3)	0.5798 (4)	0.4807 (3)	0.0749 (9)	
H3WA	0.854 (5)	0.515 (3)	0.481 (5)	0.112*	
H3WB	0.853 (5)	0.596 (6)	0.438 (4)	0.112*	
O8W	0.0051 (4)	0.4950 (3)	0.3388 (2)	0.0779 (9)	
H8WA	0.031 (5)	0.551 (4)	0.297 (3)	0.117*	
H8WB	0.071 (4)	0.453 (5)	0.385 (3)	0.117*	
O7W	−0.0580 (3)	0.4443 (3)	0.1510 (3)	0.0870 (11)	
H7WA	−0.038 (6)	0.504 (4)	0.113 (4)	0.130*	
H7WB	−0.038 (7)	0.431 (6)	0.214 (3)	0.130*	
S1	0.11520 (7)	0.68913 (6)	0.08592 (5)	0.02492 (16)	

### Atomic displacement parameters (Å^2^)

**Table:**

	*U*^11^	*U*^22^	*U*^33^	*U*^12^	*U*^13^	*U*^23^
Cd1	0.02405 (12)	0.02632 (12)	0.02400 (12)	−0.01063 (9)	0.00255 (8)	−0.01025 (9)
N1	0.0290 (13)	0.0283 (13)	0.0224 (11)	−0.0109 (11)	0.0028 (10)	−0.0084 (10)
N2	0.0339 (14)	0.0262 (13)	0.0259 (12)	−0.0116 (11)	−0.0020 (11)	−0.0082 (10)
N3	0.0268 (13)	0.0248 (12)	0.0275 (12)	−0.0105 (10)	−0.0007 (10)	−0.0099 (10)
N4	0.0286 (13)	0.0247 (12)	0.0264 (12)	−0.0095 (10)	0.0031 (10)	−0.0106 (10)
C1	0.0363 (17)	0.0362 (18)	0.0266 (15)	−0.0101 (14)	0.0056 (13)	−0.0101 (13)
C2	0.0340 (18)	0.0354 (18)	0.0404 (18)	−0.0056 (15)	0.0110 (15)	−0.0092 (15)
C3	0.047 (2)	0.0376 (19)	0.0409 (18)	−0.0159 (16)	0.0215 (16)	−0.0173 (15)
C4	0.0455 (19)	0.0401 (18)	0.0292 (15)	−0.0238 (15)	0.0157 (14)	−0.0176 (14)
C5	0.061 (2)	0.059 (2)	0.0351 (18)	−0.030 (2)	0.0143 (17)	−0.0291 (17)
C6	0.059 (2)	0.071 (3)	0.0306 (17)	−0.035 (2)	0.0067 (16)	−0.0266 (18)
C7	0.0461 (19)	0.0445 (19)	0.0247 (15)	−0.0268 (16)	0.0035 (14)	−0.0118 (14)
C8	0.055 (2)	0.055 (2)	0.0286 (16)	−0.0282 (18)	−0.0095 (16)	−0.0072 (16)
C9	0.0394 (19)	0.042 (2)	0.0403 (18)	−0.0124 (16)	−0.0122 (15)	−0.0067 (16)
C10	0.0401 (18)	0.0315 (17)	0.0334 (16)	−0.0098 (14)	−0.0055 (14)	−0.0104 (14)
C11	0.0377 (17)	0.0311 (16)	0.0211 (13)	−0.0217 (13)	0.0040 (12)	−0.0073 (12)
C12	0.0371 (16)	0.0261 (15)	0.0241 (14)	−0.0185 (13)	0.0082 (12)	−0.0097 (12)
C13	0.0348 (17)	0.0237 (15)	0.0368 (16)	−0.0074 (13)	0.0018 (14)	−0.0096 (13)
C14	0.050 (2)	0.0241 (16)	0.0453 (19)	−0.0130 (15)	−0.0041 (16)	−0.0079 (14)
C15	0.052 (2)	0.0333 (17)	0.0367 (17)	−0.0277 (16)	−0.0009 (16)	−0.0040 (14)
C16	0.0375 (17)	0.0350 (17)	0.0222 (14)	−0.0219 (14)	−0.0013 (13)	−0.0053 (12)
C17	0.0354 (18)	0.057 (2)	0.0319 (16)	−0.0303 (16)	0.0060 (14)	−0.0135 (15)
C18	0.0282 (16)	0.064 (2)	0.0262 (15)	−0.0234 (16)	0.0092 (13)	−0.0192 (15)
C19	0.0232 (15)	0.0425 (18)	0.0228 (14)	−0.0117 (13)	0.0027 (12)	−0.0145 (13)
C20	0.0288 (16)	0.047 (2)	0.0394 (18)	−0.0061 (15)	0.0071 (14)	−0.0199 (16)
C21	0.0430 (19)	0.0298 (17)	0.0460 (19)	−0.0039 (15)	0.0056 (16)	−0.0169 (15)
C22	0.0375 (17)	0.0284 (16)	0.0360 (16)	−0.0132 (14)	0.0096 (14)	−0.0131 (13)
C23	0.0262 (15)	0.0275 (15)	0.0181 (13)	−0.0108 (12)	−0.0011 (11)	−0.0076 (11)
C24	0.0262 (15)	0.0289 (15)	0.0181 (13)	−0.0146 (12)	0.0005 (11)	−0.0063 (11)
O2W	0.0377 (12)	0.0284 (12)	0.0332 (12)	−0.0104 (10)	−0.0083 (10)	−0.0089 (9)
O1W	0.0390 (12)	0.0269 (11)	0.0285 (11)	−0.0146 (9)	−0.0019 (9)	−0.0072 (9)
O1	0.0566 (15)	0.0506 (14)	0.0293 (11)	−0.0352 (12)	0.0067 (10)	−0.0138 (10)
O2	0.0272 (11)	0.0372 (12)	0.0456 (12)	−0.0072 (9)	0.0030 (10)	−0.0140 (10)
O3	0.0444 (13)	0.0238 (11)	0.0379 (12)	−0.0094 (10)	−0.0021 (10)	−0.0078 (9)
O4	0.0438 (13)	0.0506 (14)	0.0438 (12)	−0.0291 (11)	0.0066 (10)	−0.0264 (11)
O6W	0.0453 (15)	0.0450 (15)	0.0432 (15)	−0.0153 (12)	−0.0018 (11)	−0.0079 (12)
O5W	0.0495 (17)	0.0530 (17)	0.0506 (16)	−0.0061 (14)	−0.0082 (12)	−0.0123 (13)
O4W	0.069 (2)	0.094 (2)	0.0545 (16)	−0.0371 (16)	0.0106 (15)	−0.0366 (17)
O3W	0.0580 (19)	0.074 (2)	0.084 (2)	−0.0234 (16)	0.0179 (16)	−0.0151 (19)
O8W	0.078 (2)	0.080 (2)	0.067 (2)	−0.0471 (19)	0.0083 (16)	0.0079 (16)
O7W	0.0522 (18)	0.0461 (18)	0.166 (3)	−0.0217 (15)	0.038 (2)	−0.037 (2)
S1	0.0263 (4)	0.0244 (4)	0.0272 (3)	−0.0119 (3)	0.0018 (3)	−0.0094 (3)

### Geometric parameters (Å, °)

**Table:**

Cd1—O2W	2.252 (2)	C14—H14A	0.9300
Cd1—O1W	2.286 (2)	C15—C16	1.415 (4)
Cd1—N4	2.327 (2)	C15—H15A	0.9300
Cd1—N1	2.342 (2)	C16—C24	1.400 (4)
Cd1—N3	2.350 (2)	C16—C17	1.430 (4)
Cd1—N2	2.377 (2)	C17—C18	1.349 (4)
N1—C1	1.321 (4)	C17—H17A	0.9300
N1—C12	1.361 (3)	C18—C19	1.423 (4)
N2—C10	1.331 (4)	C18—H18A	0.9300
N2—C11	1.359 (4)	C19—C20	1.404 (4)
N3—C13	1.327 (3)	C19—C23	1.410 (4)
N3—C24	1.366 (4)	C20—C21	1.358 (5)
N4—C22	1.336 (3)	C20—H20A	0.9300
N4—C23	1.356 (4)	C21—C22	1.384 (4)
C1—C2	1.390 (4)	C21—H21A	0.9300
C1—H1A	0.9300	C22—H22A	0.9300
C2—C3	1.367 (4)	C23—C24	1.444 (4)
C2—H2A	0.9300	O2W—H2WA	0.83 (4)
C3—C4	1.401 (4)	O2W—H2WB	0.81 (3)
C3—H3A	0.9300	O1W—H1WA	0.84 (2)
C4—C12	1.406 (4)	O1W—H1WB	0.85 (2)
C4—C5	1.435 (4)	O1—S1	1.479 (2)
C5—C6	1.342 (5)	O2—S1	1.466 (2)
C5—H5A	0.9300	O3—S1	1.476 (2)
C6—C7	1.427 (4)	O4—S1	1.462 (2)
C6—H6A	0.9300	O6W—H6WB	0.84 (2)
C7—C8	1.404 (4)	O6W—H6WA	0.78 (3)
C7—C11	1.415 (4)	O5W—H5WA	0.77 (3)
C8—C9	1.354 (5)	O5W—H5WB	0.77 (3)
C8—H8A	0.9300	O4W—H4WA	0.87 (2)
C9—C10	1.396 (4)	O4W—H4WB	0.79 (3)
C9—H9A	0.9300	O3W—H3WA	0.76 (3)
C10—H10A	0.9300	O3W—H3WB	0.77 (3)
C11—C12	1.444 (4)	O8W—H8WA	0.85 (3)
C13—C14	1.395 (4)	O8W—H8WB	0.83 (3)
C13—H13A	0.9300	O7W—H7WA	0.83 (3)
C14—C15	1.357 (5)	O7W—H7WB	0.82 (3)
			
O2W—Cd1—O1W	82.11 (8)	C7—C11—C12	119.2 (3)
O2W—Cd1—N4	112.65 (8)	N1—C12—C4	121.8 (3)
O1W—Cd1—N4	91.28 (8)	N1—C12—C11	118.6 (2)
O2W—Cd1—N1	90.11 (8)	C4—C12—C11	119.6 (2)
O1W—Cd1—N1	106.36 (8)	N3—C13—C14	123.2 (3)
N4—Cd1—N1	153.17 (8)	N3—C13—H13A	118.4
O2W—Cd1—N3	98.54 (9)	C14—C13—H13A	118.4
O1W—Cd1—N3	161.85 (8)	C15—C14—C13	118.9 (3)
N4—Cd1—N3	71.63 (8)	C15—C14—H14A	120.5
N1—Cd1—N3	91.79 (8)	C13—C14—H14A	120.5
O2W—Cd1—N2	156.76 (9)	C14—C15—C16	120.1 (3)
O1W—Cd1—N2	89.69 (8)	C14—C15—H15A	120.0
N4—Cd1—N2	89.12 (8)	C16—C15—H15A	120.0
N1—Cd1—N2	71.32 (8)	C24—C16—C15	117.3 (3)
N3—Cd1—N2	95.96 (8)	C24—C16—C17	119.8 (3)
C1—N1—C12	118.5 (2)	C15—C16—C17	123.0 (3)
C1—N1—Cd1	125.28 (18)	C18—C17—C16	120.7 (3)
C12—N1—Cd1	116.18 (18)	C18—C17—H17A	119.6
C10—N2—C11	118.2 (2)	C16—C17—H17A	119.6
C10—N2—Cd1	126.83 (19)	C17—C18—C19	121.3 (3)
C11—N2—Cd1	114.91 (17)	C17—C18—H18A	119.3
C13—N3—C24	118.2 (3)	C19—C18—H18A	119.3
C13—N3—Cd1	126.6 (2)	C20—C19—C23	117.3 (3)
C24—N3—Cd1	114.87 (17)	C20—C19—C18	123.2 (3)
C22—N4—C23	118.1 (2)	C23—C19—C18	119.5 (3)
C22—N4—Cd1	125.5 (2)	C21—C20—C19	119.7 (3)
C23—N4—Cd1	115.83 (17)	C21—C20—H20A	120.1
N1—C1—C2	123.4 (3)	C19—C20—H20A	120.1
N1—C1—H1A	118.3	C20—C21—C22	119.7 (3)
C2—C1—H1A	118.3	C20—C21—H21A	120.2
C3—C2—C1	118.8 (3)	C22—C21—H21A	120.2
C3—C2—H2A	120.6	N4—C22—C21	122.8 (3)
C1—C2—H2A	120.6	N4—C22—H22A	118.6
C2—C3—C4	119.7 (3)	C21—C22—H22A	118.6
C2—C3—H3A	120.1	N4—C23—C19	122.4 (3)
C4—C3—H3A	120.1	N4—C23—C24	118.5 (2)
C3—C4—C12	117.8 (3)	C19—C23—C24	119.2 (3)
C3—C4—C5	122.8 (3)	N3—C24—C16	122.3 (3)
C12—C4—C5	119.4 (3)	N3—C24—C23	118.3 (2)
C6—C5—C4	121.0 (3)	C16—C24—C23	119.5 (3)
C6—C5—H5A	119.5	Cd1—O2W—H2WA	126 (3)
C4—C5—H5A	119.5	Cd1—O2W—H2WB	116 (2)
C5—C6—C7	121.7 (3)	H2WA—O2W—H2WB	106 (3)
C5—C6—H6A	119.2	Cd1—O1W—H1WA	112 (2)
C7—C6—H6A	119.2	Cd1—O1W—H1WB	124 (2)
C8—C7—C11	117.3 (3)	H1WA—O1W—H1WB	112 (3)
C8—C7—C6	123.5 (3)	H6WB—O6W—H6WA	112 (4)
C11—C7—C6	119.2 (3)	H5WA—O5W—H5WB	111 (5)
C9—C8—C7	120.5 (3)	H4WA—O4W—H4WB	108 (3)
C9—C8—H8A	119.8	H3WA—O3W—H3WB	77 (5)
C7—C8—H8A	119.8	H8WA—O8W—H8WB	104 (5)
C8—C9—C10	118.7 (3)	H7WA—O7W—H7WB	110 (6)
C8—C9—H9A	120.6	O4—S1—O2	110.75 (13)
C10—C9—H9A	120.6	O4—S1—O3	109.80 (13)
N2—C10—C9	123.3 (3)	O2—S1—O3	108.90 (13)
N2—C10—H10A	118.3	O4—S1—O1	109.62 (13)
C9—C10—H10A	118.3	O2—S1—O1	109.88 (13)
N2—C11—C7	121.9 (3)	O3—S1—O1	107.83 (13)
N2—C11—C12	118.9 (2)		
			
O2W—Cd1—N1—C1	−14.3 (2)	C10—N2—C11—C7	−0.3 (4)
O1W—Cd1—N1—C1	−96.1 (2)	Cd1—N2—C11—C7	−179.2 (2)
N4—Cd1—N1—C1	134.7 (2)	C10—N2—C11—C12	−179.4 (3)
N3—Cd1—N1—C1	84.3 (2)	Cd1—N2—C11—C12	1.6 (3)
N2—Cd1—N1—C1	180.0 (3)	C8—C7—C11—N2	−0.7 (4)
O2W—Cd1—N1—C12	169.1 (2)	C6—C7—C11—N2	179.7 (3)
O1W—Cd1—N1—C12	87.3 (2)	C8—C7—C11—C12	178.5 (3)
N4—Cd1—N1—C12	−41.9 (3)	C6—C7—C11—C12	−1.2 (4)
N3—Cd1—N1—C12	−92.36 (19)	C1—N1—C12—C4	−0.1 (4)
N2—Cd1—N1—C12	3.33 (18)	Cd1—N1—C12—C4	176.7 (2)
O2W—Cd1—N2—C10	140.1 (3)	C1—N1—C12—C11	179.3 (3)
O1W—Cd1—N2—C10	71.2 (3)	Cd1—N1—C12—C11	−3.8 (3)
N4—Cd1—N2—C10	−20.1 (3)	C3—C4—C12—N1	−0.5 (4)
N1—Cd1—N2—C10	178.6 (3)	C5—C4—C12—N1	178.5 (3)
N3—Cd1—N2—C10	−91.5 (3)	C3—C4—C12—C11	−180.0 (3)
O2W—Cd1—N2—C11	−41.1 (3)	C5—C4—C12—C11	−0.9 (4)
O1W—Cd1—N2—C11	−110.0 (2)	N2—C11—C12—N1	1.4 (4)
N4—Cd1—N2—C11	158.7 (2)	C7—C11—C12—N1	−177.7 (3)
N1—Cd1—N2—C11	−2.57 (19)	N2—C11—C12—C4	−179.1 (3)
N3—Cd1—N2—C11	87.3 (2)	C7—C11—C12—C4	1.7 (4)
O2W—Cd1—N3—C13	67.9 (2)	C24—N3—C13—C14	−0.6 (4)
O1W—Cd1—N3—C13	158.6 (2)	Cd1—N3—C13—C14	172.6 (2)
N4—Cd1—N3—C13	179.0 (2)	N3—C13—C14—C15	−0.2 (5)
N1—Cd1—N3—C13	−22.5 (2)	C13—C14—C15—C16	0.4 (4)
N2—Cd1—N3—C13	−93.9 (2)	C14—C15—C16—C24	0.1 (4)
O2W—Cd1—N3—C24	−118.70 (17)	C14—C15—C16—C17	−179.7 (3)
O1W—Cd1—N3—C24	−28.0 (3)	C24—C16—C17—C18	−0.2 (4)
N4—Cd1—N3—C24	−7.57 (16)	C15—C16—C17—C18	179.6 (3)
N1—Cd1—N3—C24	150.92 (17)	C16—C17—C18—C19	0.1 (4)
N2—Cd1—N3—C24	79.52 (18)	C17—C18—C19—C20	−179.7 (3)
O2W—Cd1—N4—C22	−88.6 (2)	C17—C18—C19—C23	0.4 (4)
O1W—Cd1—N4—C22	−6.6 (2)	C23—C19—C20—C21	0.7 (4)
N1—Cd1—N4—C22	125.4 (2)	C18—C19—C20—C21	−179.2 (3)
N3—Cd1—N4—C22	179.7 (2)	C19—C20—C21—C22	0.1 (5)
N2—Cd1—N4—C22	83.1 (2)	C23—N4—C22—C21	1.5 (4)
O2W—Cd1—N4—C23	100.12 (18)	Cd1—N4—C22—C21	−169.6 (2)
O1W—Cd1—N4—C23	−177.90 (18)	C20—C21—C22—N4	−1.3 (5)
N1—Cd1—N4—C23	−46.0 (3)	C22—N4—C23—C19	−0.6 (4)
N3—Cd1—N4—C23	8.35 (17)	Cd1—N4—C23—C19	171.41 (18)
N2—Cd1—N4—C23	−88.23 (18)	C22—N4—C23—C24	179.6 (2)
C12—N1—C1—C2	0.2 (4)	Cd1—N4—C23—C24	−8.4 (3)
Cd1—N1—C1—C2	−176.4 (2)	C20—C19—C23—N4	−0.5 (4)
N1—C1—C2—C3	0.5 (5)	C18—C19—C23—N4	179.4 (2)
C1—C2—C3—C4	−1.1 (5)	C20—C19—C23—C24	179.3 (2)
C2—C3—C4—C12	1.1 (5)	C18—C19—C23—C24	−0.8 (4)
C2—C3—C4—C5	−177.9 (3)	C13—N3—C24—C16	1.1 (4)
C3—C4—C5—C6	178.5 (3)	Cd1—N3—C24—C16	−172.87 (19)
C12—C4—C5—C6	−0.5 (5)	C13—N3—C24—C23	−179.8 (2)
C4—C5—C6—C7	1.1 (5)	Cd1—N3—C24—C23	6.3 (3)
C5—C6—C7—C8	−179.9 (4)	C15—C16—C24—N3	−0.9 (4)
C5—C6—C7—C11	−0.3 (5)	C17—C16—C24—N3	178.9 (2)
C11—C7—C8—C9	1.4 (5)	C15—C16—C24—C23	180.0 (2)
C6—C7—C8—C9	−179.0 (3)	C17—C16—C24—C23	−0.2 (4)
C7—C8—C9—C10	−1.2 (5)	N4—C23—C24—N3	1.4 (3)
C11—N2—C10—C9	0.5 (5)	C19—C23—C24—N3	−178.4 (2)
Cd1—N2—C10—C9	179.3 (2)	N4—C23—C24—C16	−179.5 (2)
C8—C9—C10—N2	0.2 (5)	C19—C23—C24—C16	0.7 (4)

### Hydrogen-bond geometry (Å, °)

**Table:**

*D*—H···*A*	*D*—H	H···*A*	*D*···*A*	*D*—H···*A*
O1W—H1WA···O1	0.84 (2)	1.84 (2)	2.657 (3)	163 (3)
O2W—H2WB···O3	0.81 (3)	1.92 (3)	2.718 (3)	169 (4)
O1W—H1WB···O6W	0.85 (2)	1.91 (2)	2.739 (3)	163 (3)
O5W—H5WA···O2	0.77 (3)	2.08 (3)	2.816 (3)	161 (5)
O8W—H8WA···O1	0.85 (3)	1.88 (3)	2.729 (4)	175 (5)
O4W—H4WA···O2	0.87 (2)	1.99 (3)	2.805 (3)	157 (4)
O6W—H6WB···O5W	0.84 (2)	2.04 (3)	2.830 (4)	158 (4)
O7W—H7WA···O4	0.83 (3)	1.96 (3)	2.791 (4)	172 (6)
O7W—H7WB···O8W	0.82 (3)	2.16 (4)	2.901 (5)	151 (6)
O2W—H2WA···O3^i^	0.83 (4)	1.86 (4)	2.671 (3)	164 (3)
O3W—H3WB···O8W^ii^	0.77 (3)	2.29 (5)	2.907 (5)	137 (6)
O5W—H5WB···O4W^iii^	0.77 (3)	2.07 (3)	2.829 (4)	174 (5)
O6W—H6WA···O3W^iv^	0.78 (3)	2.03 (3)	2.773 (5)	160 (4)
O4W—H4WB···O7W^v^	0.79 (3)	2.03 (3)	2.806 (4)	168 (5)
O8W—H8WB···O3W^iv^	0.83 (3)	2.09 (3)	2.851 (5)	154 (5)

Symmetry codes: (i) −*x*, −*y*+2, −*z*; (ii) *x*+1, *y*, *z*; (iii) −*x*+1, −*y*+1, −*z*; (iv) −*x*+1, −*y*+1, −*z*+1; (v) −*x*, −*y*+1, −*z*.
